# Genomic Characterization of MDR* Escherichia coli* Harboring* bla*_OXA-48_ on the IncL/M-type Plasmid Isolated from Blood Stream Infection

**DOI:** 10.1155/2018/3036143

**Published:** 2018-06-28

**Authors:** S. Alousi, T. Salloum, H. Arabaghian, G. M. Matar, G. F. Araj, S. T. Tokajian

**Affiliations:** ^1^Department of Natural Sciences, School of Arts & Sciences, Lebanese American University, Byblos, Lebanon; ^2^Center for Infectious Diseases Research, American University of Beirut Medical Center, Beirut, Lebanon; ^3^Department of Experimental Pathology, Immunology & Microbiology, Faculty of Medicine, American University of Beirut Medical Center, Beirut, Lebanon; ^4^Department of Pathology & Laboratory Medicine, Faculty of Medicine, American University of Beirut Medical Center, Beirut, Lebanon

## Abstract

*Escherichia coli* is responsible for a wide variety of community and hospital acquired extraintestinal infections, and the emergence of ESBL resistant isolates is a major clinical concern. In this study, we characterized the genomic attributes of an OXA-48 and CTX-M-3 producing* E. coli *EC-IMP153. Whole-genome initial assembly produced 146 contigs with a combined 5,504,170 bp in size and a G+C content of 50.5%. wgSNPs-based phylogenetic comparison with 36 publically available genomes was also performed. Comprehensive genomic analysis showed that EC-IMP153 belonged to sequence type ST-405 and harbored several resistance determinants including the *β*-lactam resistance genes *bla*_OXA-48_, *bla*_CTX-M-3_, *bla*_TEM-1B_, *bla*_OXA-1_, and *bla*_CMY-70_, aminoglycoside* fyuA *and* aac(3)IId*, tetracycline* tet(A) *and* tet(R)*, and fluoroquinolone* gyrA, parC, *and* mfd* resistance determinants. Plasmids with the following incompatibility groups were detected* in silico* and confirmed using PBRT: IncI1-*α*, IncL, IncW, Col (BS512), and IncF. To our knowledge this is the first in-depth genomic analysis of an OXA-48 producing* E. coli* ST-405 isolated from a patient in Lebanon and linked to a blood stream infection. Continuous monitoring is necessary to better understand the continued diffusion of such pathogens, especially in view of the population movements triggered by unrest in the Middle East.

## 1. Introduction

*Escherichia coli* is responsible for a wide variety of community and hospital acquired extraintestinal infections. Extraintestinal pathogenic* E. coli* (ExPEC) is widely known to cause bloodstream, urinary and respiratory tract, cerebrospinal fluid, and peritoneum infections [1, 2]. Successful treatment of infections caused by pathogenic* E. coli* could be achieved through the use of *β*-lactams. In recent years, there has been an evident increase in *β*-lactamases production, including extended spectrum *β*-lactamase (ESBL), plasmid-mediated AmpC *β*-lactamase (e.g., CMY), and carbapenemases produced by ExPEC [3]. Three most significant classes of carbapenemases are the class A (e.g., KPC), class B also known as metallo *β*-lactamases (e.g., NDM), and class D OXA-types (e.g., OXA-48) [3].

The OXA-48 *β*-lactamase was first identified in* Enterobacteriaceae* in Turkey in 2001, with OXA-48 positive* Enterobacteriaceae *belonging to ESBL producers and nonproducers [4]. Subsequently* E. coli* producing OXA-48-like variants were reported in different countries around the world [5] including Turkey, Belgium, France, and Lebanon [6]. Generally, OXA-48 producing isolates are multidrug-resistant (MDR) and are able to hydrolyze antimicrobial agents at different levels, exhibiting high hydrolyzing activity towards penicillins, low activity against carbapenems, and sparing broad-spectrum cephalosporins, and are not susceptible to *β*-lactamase inhibitors [7].

Genes encoding for *β*-lactamases are generally located on mobile genetic elements such as transposons, plasmids, and integrons [8]. The spread of the *bla*_OXA-48_ gene is regularly linked to the dissemination of the 62-kb IncL/M-type plasmid, with most of the OXA-48-positive* Enterobacteriaceae* harboring this specific type of plasmid [9]. Previously Matar et al. (2008) [6] reported the emergence of carbapenemase OXA-48 in Lebanon in the periods 2008-2010 with* Klebsiella pneumoniae* being the major OXA-48 producing* Enterobacteriaceae. *In 2012,* E. coli* strains isolated from Lebanon represented 73% of clinical producers of OXA-48 [10].

In this study, using genome sequencing we aimed at investigating and characterizing the genetic background and horizontally transferable MDR resistance determinants in* E. coli* EC-IMP153. Particular interest was given to the IncL/M-type plasmid carrying *bla*_OXA-48_ and to IncFII plasmid having *bla*_TEM-1B_, *bla*_CTXM-3_, *bla*_OXA-1_,* tet(A)*, and* tet(R)*. The genetic environment of the plasmids carried by EC-IMP153 revealed the coexistence of multiple resistance genes on the same plasmid. Additionally, MLST, wgSNPs, and comparative genome analysis were considered to investigate the phylogeny of the isolate.

## 2. Materials and Methods

### 2.1. Sample Collection

The isolate was recovered from the blood samples of a patient admitted to the American University of Beirut Medical Center (AUBMC) in 2010.

### 2.2. Antimicrobial Susceptibility Testing

Antimicrobial susceptibility profile of EC-IMP153 isolate was screened through the disk diffusion method on Muller-Hinton agar for the following antibiotics: ampicillin, gentamicin, piperacillin, piperacillin-tazobactam, amoxicillin-clavulanic acid, cefepime, ceftriaxone, cefuroxime, cefotaxime, ceftazidime, aztreonam, imipenem, meropenem, ertapenem, colistin, trimethoprim-sulfamethoxazole, tetracycline, and ticaracillin.* E. coli* ATCC 25922 was used for routine quality control. Results were interpreted according to the Clinical Laboratory Standards Institutes (CLSI) criteria [11]. Screening tests for ESBL production were performed through double-disk synergy test (cefotaxime and ceftazidime disks with and without clavulanic acid) following the CLSI criteria [11].* K. pneumoniae* ATCC 700603 was used as positive control for ESBL production.

### 2.3. DNA Isolation

Extraction of bacterial DNA was performed, after growing on tryptone soy broth overnight at 37°C, using the NucleoSpin® Tissue Kit (Macherey-Nagel, Germany) according to the manufacturer's protocol.

### 2.4. Genome Sequencing

Library preparation was done using genomic DNA (gDNA). Bioruptor® NGS was used to sonicate 1 *μ*g of sample DNA. The resulting sheared DNA was used as input for library preparation using the Illumina TruSeq DNA library preparation kit (Illumina). The gDNA was further subjected to end-repair, A-tailing, ligation of adaptors as recommended by the manufacturer. Fragments between 500 and 1000 bp were selected using the Pippin Prep™ DNA size selection system (Sage Science). qPCR was used to quantify the resulting library in triplicate at 1:1000 and using the Kapa library quantification kit (Kapa Biosystems, Woburn, MA, USA), following the manufacturer's instructions. The resultant library size was assessed using an Agilent Bioanalyzer with the High Sensitivity DNA Kit. The library was multiplexed, clustered, and sequenced on an Illumina MiSeq with paired-end 500 cycles protocol to read a length of 250 bp.

### 2.5. Genome Assembly

Genome assembly was performed* de novo* using A5 with default parameters [14]. This pipeline automates the processes through several steps: read quality filtering and error correction, contig assembly, permissive draft scaffolding, misassembly detection, and conservative scaffolding.

### 2.6. Genome Annotation and Analysis

The assembled draft genome was annotated using RAST. The RAST server identifies protein-encoding genes, rRNA and tRNA, and predicts the different subsystems within the genome [15]. The Antibiotic Resistance Database (ARDB) [16] and ResFinder server v2.1 were used to identify resistance genes using a threshold of 90% identity (ID) [17]. The multilocus sequence type (MLST) was determined using two different MLST schemes available on CGE server, i.e., MLST1; the Achtman scheme and MLST2; the Pasteur scheme. The presence of plasmids and corresponding sequence types (STs) were determined using both* in silico* PlasmidFinder 1.3 server [18] and pMLST available on CGE. IS-finder was used to identify insertion sequences (ISs) and identify IS-families [19]. PLACNETw separated chromosomal genome from accessory plasmid genomes, based on paired-end reads assembly [20].

### 2.7. Plasmid DNA Extraction

For plasmid extraction the isolate was inoculated on Luria Bertani broth and incubated overnight at 37°C. The QIAprep Spin Miniprep Kit (Qiagen, Hilden, Germany) was used according to the manufacturer's instructions.

### 2.8. Identification of Resistance Genes

The *β*-lactamase gene *bla*_OXA-48_ was traced by PCR amplification, on both crude genomic DNA and bacterial plasmid DNA, using the following set of primers: OXA-48A (5′-TTGGTGGCATCGATTATCGG-3′) and OXA-48B (5′-GAGCACTTCTTTTGTGATGGC-3′) as described by Aktas et al. (2008) [12]. PCR amplicons were electrophoresed on 1.5% agarose gels. Automated sequencing was performed on ABI 3500 DNA analyzer using the Big Dye system (Applied Biosystems Foster City, CA, USA). *bla*_OXA-48_ gene sequence was compared with online sequences using the BLASTn. Similarly, *bla*_AmpC_ was amplified through PCR on both crude genomic DNA and plasmid DNA using the following set of primers: AmpC-F: (5′-ATGATGAAAAAATCGTTATGC-3′) and AmpC-R: (5′-TTGCAGCTTTTCAAGAATGCGC-3′) [13] (Table 1).

The locations and genetic environment of *bla*_OXA-48_ and *bla*_CTXM-3_ were determined through sequence alignment on BioNumerics v7.6.1 beta software (Applied Maths, Sint-Martens-Latem, Belgium). The sequence of IncFII plasmid was extracted and the genetic environment of resistance genes *bla*_TEM-1B_, *bla*_CTXM-3_, and *bla*_OXA-1_ was annotated using RAST and IS-finder.

Fluoroquinolone resistance-determining regions (QRDR) of* gyrA* and* parC* were compared to the amino acid sequence of that in* E. coli* K-12 (GenBank accession no. NC_000913). Protein alignment was performed on EMBOSS Needle tool available on EMBL-EBI website (http://www.ebi.ac.uk/Tools/).

### 2.9. Plasmid Typing

EC-IMP153 isolate genomic DNA was subjected to PCR-based replicon typing analysis (PBRT) to determine plasmid incompatibility groups as described by Carattoli et al. (2011) [21]. Eight multiplex PCRs were performed for the amplification of 28 replicons: L/M, N, FIA, FIB, FIC, FII, FIIS, FIIK, FIB-M, W, Y, P, A/C, T, K, U, R, B/O, HI1, HI2, I1, I2, X1, X2, and HIB-M representative of major plasmid incompatibility groups and replicase genes that are typically found on resistance plasmids circulating among* Enterobacteriaceae* [21, 22].

### 2.10. Genome Comparative Analysis

*E.coli* EC-IMP153 was compared with the following genomes of ST-405* E. coli* isolates: 50579417 (Accession #: NZ_LNHL00000000) OXA-48 producing* E. coli* isolated from a patient in Norway with a travel history to Thailand and used as a reference strain [23],* E.coli* LAU-EC4 (Accession #: AYOP0100000000), and* E.coli* LAU-EC5 (Accession #: AYOG0100000000) isolated from Lebanon [24] and circular visualization was constructed using CGViewer [25].

### 2.11. wgSNPs Phylogenetic Analysis

The genome was compared against the GenBank Nucleotide database using BLASTN (https://blast.ncbi.nlm.nih.gov/Blast.cgi) to identify the closest relative genomes available on the database (Supplementary Table 1: reference genomes accession numbers). BioNumerics v7.6.1 beta software (Applied Maths, Sint-Martens-Latem, Belgium) was used to align* E. coli* genomes against reference genome* E. coli* 50579417. SNP-calling was performed by mapping the paired-end reads of isolate EC-IMP153 and 34 assembled* E. coli* genomes obtained from NCBI to the reference genome of 50579417* E. coli* strain.* K. pneumoniae *genome was used as an outgroup. SNPs were deduced through strict SNP filtering for each genome sequence using BioNumerics Chromosome Comparisons module. A neighbor-joining (NJ) tree was generated in BioNumerics by using mutation filtering module, filtered from wgSNP data input.

### 2.12. Nucleotide Sequence Accession Number

The whole-genome shotgun project has been deposited at DDBJ/EMBL/GenBank under the accession number $\underline{LJOJ000000000}$. The versions described here is LJOJ01000000.

## 3. Results

### 3.1. Antimicrobial Resistance Patterns

*E. coli* EC-IMP153 was found to be resistant to tetracycline, ticaracillin, gentamycin, ampicillin, piperacillin, piperacillin/tazobactam, amoxicillin/clavulanic acid, cefepime, ceftriaxone, cefuroxime, azetronam, ceftazidime, and cefotaxime. It additionally showed intermediate resistance to trimethoprime/sulfamethoxazole but was sensitive to ertapenem, colistin, meropenem, and imipenem.

### 3.2. Genome Characterization

**Figure 1:**
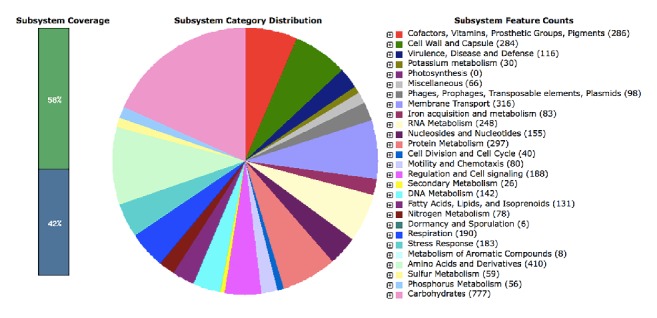
Subsystem categorical distribution in* E. coli* EC-IMP153.

*E. coli* EC-IMP153 genome consisted of 5,504,170 bp and a G+C content of 50.5% in 146 contigs, with 5,384 coding sequences (CDS) and a total of 116 predicted RNAs. RAST also distinguished genes encoding carbohydrate metabolism (777), amino acids and derivatives (410), cofactors, vitamins, prosthetic groups, pigments (286), cell wall and capsule (284), and virulence disease and defense (116) (Figure 1)

### 3.3. Isolate Typing

*In silico *(MLST1) based analysis using seven house-keeping genes (*adk, fumC, gyrB, icd, mdh, purA, *and* recA*) classified EC-IMP153 as belonging to ST-405 based on Achtman scheme and ST-44 based on the Pasteur scheme. The serotype was predicted to be O102:H6.

### 3.4. Mobile Genetic Elements

**Figure 2:**
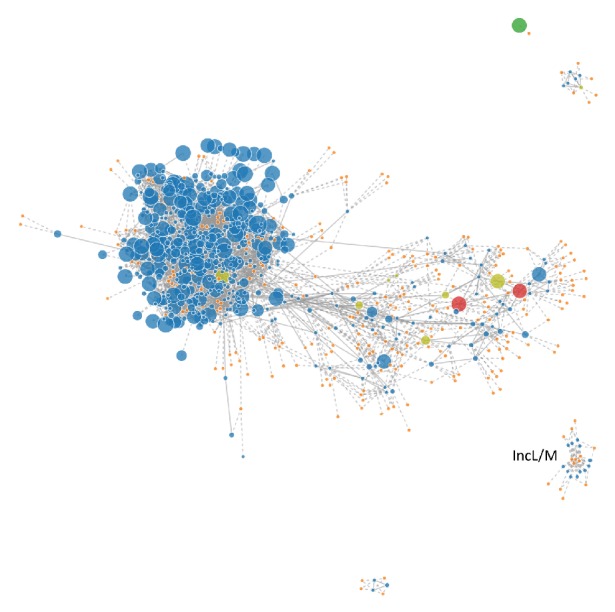
PLACNETw caption of plasmid IncL/M. Separate representation of chromosomal genome and IncL/M plasmid in* E. coli* EC-IMP153.

EC-IMP153 was positive for the plasmids with the following incompatibility groups: IncF (IncFII, IncFIA, and IncFIB), IncI1-*α*, and Col (BS512)* in silico* pMLST analysis using the FAB (FII:FIA:FIB) typing scheme for IncF plasmids, showing that the replicons belonged to the F31:A4:B1 type. Plasmid profiling by PBRT confirmed the presence of IncFIA, IncFII, IncI1-*α*, IncL, and IncW type plasmids. PLACNETw network (Figure 2) revealed the presence of fragmented, short read contigs corresponding to the IncL/M plasmid that specifically carried the *bla*_OXA-48_ gene.

IS-Finder identified 158 insertion sequences (ISs) and 180 open-reading frames (ORFs) related to ISs. Important ISs families included IS*1 *family (IS*1A*, IS*1B*, IS*1D*, IS*1G*, IS*1H*, IS*1R*, IS*1S*, IS*1SD*, IS*1X2*, and IS*1X4*), IS*1380* family (IS*Ecp1* and IS*Ec9*) with IS*Ecp1* detected upstream of *bla*_CTX-M-3_ gene, IS*3* family (IS*103*, IS*1203*, IS*1397*, IS*150*, IS*2*, IS*911*, IS*Ec27*, IS*Ec52*, IS*Kpn8*, IS*Sd1*, and IS*SFl10*), and IS*5* family (IS*5*, IS*5D*, and IS*Kpn26*).

### 3.5. Identification of Antibiotic Resistance Genes

**Figure 3:**
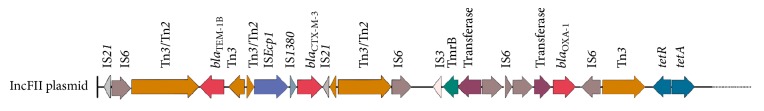
IncFII plasmid genomic environment. Structure of the *bla*_TEM-1B_, *bla*_CTX-M-3_, and *bla*_OXA-1_ genes on IncFII plasmid in* E. coli* EC-IMP153.

*bla*_TEM-1B_, *bla*_CTXM-3_, and *bla*_OXA-1_, in addition to the gene encoding a tetracycline efflux protein* tet(A)* and* tet(R)*, coexisted on the same plasmid IncFII. *bla*_CTX-M-3_ was associated with a Tn*2*/Tn*3* hybrid with an upstream IS*Ecp1*. The downstream end of Tn*2* was truncated by IS*21.* This multiresistance region (MRR) also included* tet(A), tet(R)* genes and *bla*_OXA-1_ (Figure 3).

*bla*_OXA-48_ gene specific PCR assay was additionally used on a plasmid extract which confirmed that the isolate was *bla*_OXA-48_ carrier. Sequence analysis was done and the sequence matched with the publically available NCBI sequences for the *bla*_OXA-48_ gene. *bla*_OXA-48_ was the only resistance determinant found on IncL plasmid associated with Tn*1999 *transposon. Only one copy of IS*1R *was found upstream of the gene and* lysR* being located downstream.

Aminoglycoside (*aac(3)-IId)*, macrolide (*mphA)*, and tetracycline (*tet(A)* and* tet(R)) *resistance genes were detected. CARD Resistance Gene Identifier (RGI) further revealed the presence of other resistance determinants including *bla*_CMY-70_ (*β*-lactam resistance) and* mfd*,* gyrA,* and* parC *(fluroquinolone resistance). Mutations in* gyrA* and* parC* genes leading to a single amino acid substitution in* parC* (S80I) and double substitutions in* gyrA* (S83L and D87N) were also detected.

### 3.6. Genomic Comparison

**Figure 4:**
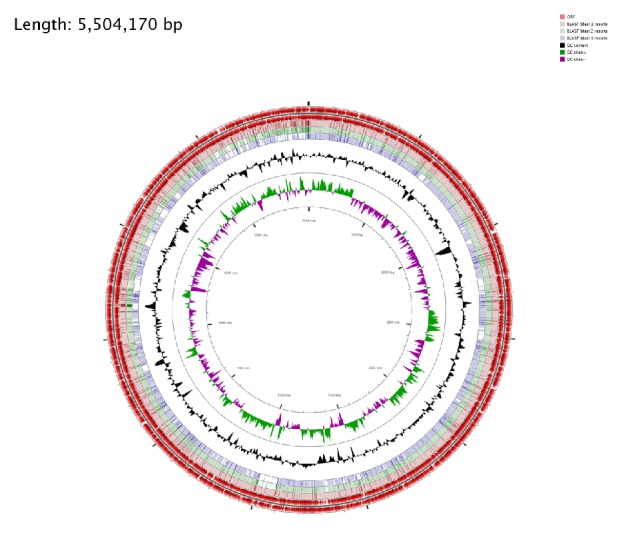
Circular genome representation of EC-IMP153 compared with* E. coli* 50579417 and* E. coli* K-12 MG1665. The outermost ring: EC-IMP153 open-reading frames (ORF) on both forward and reverse strands (red),* E. coli* blast 1 results for EC-IMP153 (light pink),* E. coli* blast 2 results 50579417 (green),* E. coli* blast 3 results K-12 MG1665 (purple) representing the positions covered by the BLASTN alignment, G+C content (black), G+C positive skew (green), and G+C negative skew (purple). Image created using CGview Server.

**Figure 5:**
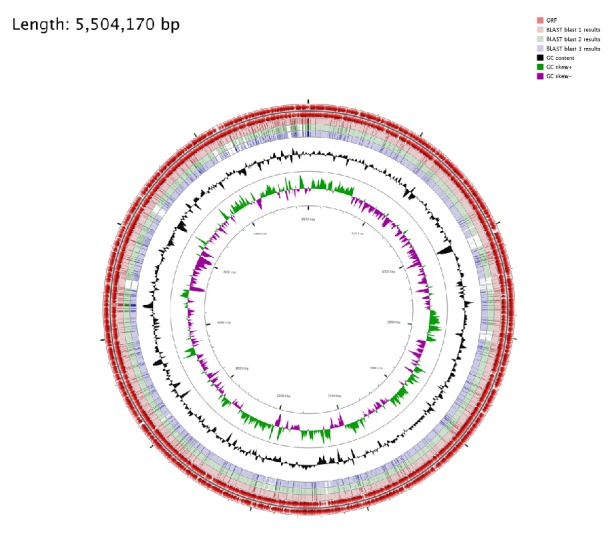
Circular genome representation of EC-IMP153 compared with* E. coli* LAU-EC4 and* E. coli* LAU-EC5. The outermost ring: EC-IMP153 open-reading frames (ORF) on both forward and reverse strands (red),* E. coli* blast 1 results for EC-IMP153 (light pink),* E. coli* blast 2 results LAU-EC4 (green),* E. coli* blast 3 results LAU-EC5 (purple) representing the positions covered by the BLASTN alignment, G+C content (black), G+C positive skew (green), and G+C negative skew (purple). Image created using CGview Server.

Circular visualization and comparison of the genomic sequences were generated using CGViewer server.* E. coli* EC-IMP153 was compared with 50579417 and* E. coli* K-12 MG1665 (Figure 4) and with LAU-EC4 and LAU-EC5 (Figure 5).

### 3.7. SNPs Based Phylogenetic Analysis

**Figure 6:**
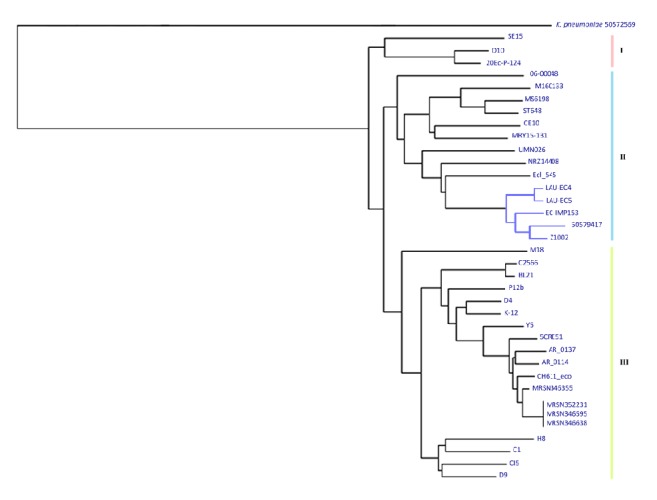
SNPs based phylogenetic tree. EC-IMP153 (LJOJ01000000), 50579417 (NZ_LNHL00000000), OXA-48 producing* E. coli* isolated from Norway, LAU-EC4 (AYOP0100000000), LAU-EC5 (AYOG0100000000) isolated from Lebanon, and Z1002 (AE005174) clustering in one clade.

wgSNPs-based phylogenetic analysis of EC-IMP153 with 36 reference genomes downloaded from NCBI separated the isolates into three major clades (I, II, and III). Clade II was further subdivided into four subclades. Four ST-405 isolates including EC-IMP153, two CTX-M-15, and OXA-1 producing isolates (LAU-EC4 and LAU-EC5) [26],* E. coli* 50579417 harboring CTX-M-1 and OXA-48 genes isolated from Norway [24], and* E. coli* Z1002 belonging to ST-11 all clustered together. The distribution of the isolates correlated mainly with the area of isolation and ST (Figure 6).

## 4. Discussion

This study, to the best of our knowledge, is the first in-depth comparative genomic analysis of ST-405 OXA-48 producing MDR* E. coli*, linked to bacteremia isolated from a patient in Lebanon. Bloodstream infections (BSIs) caused by* E. coli* have been associated with prolonged hospital stay [25]. ST-405 is classified as one of the important ExPEC lineages [27] and has been involved in the spread of genes encoding ESBLs, cephamycinases, and carbapenemases [28, 29]. Previous surveillance studies conducted across European countries and North and South America have shown that around 20 to 45% of ExPEC were resistant to the first line of antibiotics including cephalosporins, fluoroquinolones, and trimethoprim-sulfamethoxazole [30]. EC-IMP153 was of serotype O102:H6, previously linked to* E. coli* ST-405 strains in France [31], and to ST964 (O102:H6) strains in Norway [32].

EC-IMP153 was positive for the *bla*_OXA-48_, an important carbapenem-resistant determinant. The OXA carbapenemases are presented by different class D OXA enzymes such as OXA-23-like, OXA-24-like, OXA-48, OXA-51-like, and OXA-58-like [33]. Poirel et al. (2011) [34] linked the distribution of OXA-48 producers, particularly in the Mediterranean region and in Western Europe, to the spread of the IncL/M-type plasmid, which carries the *bla*_OXA-48_ gene as the only resistant determinant. This was in harmony with our results, and EC-IMP153 was positive for *bla*_OXA-48_ and carried the IncL plasmid type as confirmed by WGS, PBRT testing, and PLACNETw. Earlier studies indicated that plasmids from different countries around the world carrying the OXA-48 gene shared related features; being self-conjugative, similar in size and not having other resistance determinants [22, 35]. Sequence analysis of IncL/M plasmid showed that the *bla*_OXA-48_ gene is bracketed by two copies of IS*1999*, giving rise to a composite transposon Tn*1999 *[4].

Furthermore, detailed analysis of resistance determinants revealed that EC-IMP153 was positive for *bla*_CTX-M-3_, *bla*_TEM-1B_, *bla*_CMY-70_,* tet(A)*,* tet(R)*, and* aac(3)-lld*. The CTX-M enzymes have been the most predominant among* Enterobacteriaceae* [36]. CTX-M group 1 is represented by *bla*_CTX-M-1_, *bla*_CTX-M-3_, and *bla*_CTX-M-15_, with *bla*_CTX-M-1_ and *bla*_CTX-M-15_ being associated with plasmids belonging to incompatibility groups IncI1, IncFII, and IncN [37], most of which were detected in this study. *bla*_CTX-M-3_ and *bla*_OXA-1_ were found coexisting on IncFII plasmid in EC-IMP153. IncFII originated from* Kluyvera ascorbata* through IS*Ecp1* that functions as a promoter for the expression of adjacent genes [38, 39]. Although IS*Ecp1* is bracketed by two 14-bp inverted repeats (IRL and IRR), it mobilizes adjacent regions through IRL and alternative similar sequences forming 5-bp direct repeats (DR) [38]. Studying the genetic content of IncF plasmid in EC-IM1P53 revealed that the transposition unit carrying *bla*_CTX-M-3_ is inserted in the Tn*2*/Tn*3 *hybrid, which also carries *bla*_TEM-1B_ and was in harmony with previous reports [40, 41]. This model was also detected in *bla*_CTX-M-15_ positive* E. coli* isolates; *bla*_CTX-M-3_ is the progenitor of *bla*_CTX-M-15_ differing by a single amino acid substitution Asp-240 to Gly [38] and based on Ambler numbering [42]. Additionally, as previously reported, the downstream end of Tn*2* was truncated by IS*21* [43]. The association of Tn*2* with ISs may also be important for the spread of *bla*_CTX-M_, especially as complete or partial copies of Tn*2 *are frequently found in MRRs and on plasmids [40].

The *bla*_CMY-2_ gene was first detected in 1990 [44]. Plasmid-mediated AmpC genes express resistance to broad-spectrum cephalosporins [45]. Latest reports highlighted new variants of CMY, CMY-70, which was detected in this study and was registered in Lahey database (http://www.lahey.org/Studies/other.asp#table1) [46]. On the other hand, sequence comparison at the amino acid level for* gyrA* and* parC *genes detected in EC-IMP153 revealed the presence of the single substitution S80I in ParC [47] and (S83L and D87N) in gyrA, conferring resistance to fluoroquinolones [48].

Replicon sequence typing of the detected IncF plasmid in EC-IMP153 revealed that it belonged to pMLST F31:A4:B1. This ST was found to be a common pMLST in* E. coli* ST617, ST131, and ST44.* E. coli* EC-IMP153 had other different virulence determinants, including genes linked to different STs (ST69, ST393, and ST405), associated with biofilm formation (*fimH, papC and papG, fyuA or kpsMT II*), which could favor persistence [49]. In addition, Col (BS512) plasmid detected in EC-IMP153 is known as the* Shigella boydii *plasmid pBS512 with replicon type FIIA and being categorized as invasive plasmid with relation to type three secretion system (T3SS) [50].

SNPs were considered to investigate the phylogeny and detect possible clonal links between EC-IMP153 and 36 other isolates. The wgSNPs-based phylogenetic analysis distributed the isolates depending on both the isolation site and STs. Similar to other reports, isolates clustering together on the same clade were of the same phylogenetic group [51] but with different resistance profiles [52]. Visual representation of circular genomes showed the presence of unique genes found in EC-IMP153 but not in 50579417, LAU-EC4, LAU-EC5, and K-12 MG1665.

## 5. Conclusion

In this study, we identified the resistome of IncF plasmids and the genetic environments surrounding *bla*_OXA-48_ producing* E. coli* isolated from Lebanon. The isolate was a MDR that coproduced ESBLs and other plasmid-mediated resistance determinants. Our findings suggest that many IncF as well other plasmids have incorporated into the OXA-48 positive isolate. Because of limited therapeutic options and higher mortality caused by these carbapenem-resistant* Enterobacteriaceae*, continuous surveillance and molecular characterization of OXA-48 producers are needed to shed light upon all of the transmission pathways.

## Figures and Tables

**Table 1:** Primers sequences of *bla*_OXA-48_ and *bla*_AmpC_ and PCR conditions used for the amplification along with target size in (bp).

**Primer name**	**Target**	**Sequence (5**′**-3**′**)**	**Size (bp)**	**T [**°**C]**	**Reference**
OXA-48A	*bla*_OXA-48_	TTGGTGGCATCGATTATCGG	743	60	[12]
OXA-48B		GAGCACTTCTTTTGTGATGGC			

AmpC-F	*bla*_AmpC_	ATGATGAAAAAATCGTTATGC	1,143	56	[13]
AmpC-R		TTGCAGCTTTTCAAGAATGCGC			

## Data Availability

Whole Genome Shotgun project of* Escherichia coli* isolate EC-IMP153 has been deposited at DDBJ/EMBL/GenBank under the accession number LJOJ00000000. The version described in this paper is version LJOJ00000000.

## Supplementary Materials

Supplementary Materials Reference genomes accession numbers retrieved from GenBank Nucleotide database.
