# Supplementary material for: Genomic Characterization of MDR Escherichia coli Harboring blaOXA-48 on the IncL/M-type Plasmid Isolated from Blood Stream Infection
Source: Biomed Res Int. 2018 Jun 28;2018:3036143. doi: 10.1155/2018/3036143 (PMC6046176; doi:10.1155/2018/3036143)
Supplement: Supplementary Materials — Reference genomes accession numbers retrieved from GenBank Nucleotide database. [file 3036143.f1.docx]

**Biomed Research/Microbiology**

Supplementary Material

**Genomic Characterization of MDR *Escherichia coli* Harboring *bla*_OXA-48_ on the IncL/M-type Plasmid Isolated from Blood Stream Infection**

S. Alousi^1^, T. Salloum^1^, H. Arabaghian^1^, G. M. Matar^2^, G. Araj^3^, S. T. Tokajian^1*^

^1^Department of Natural Sciences, School of Arts and Sciences, Lebanese American University, Byblos, Lebanon.

^2^ Department of Experimental Pathology, Immunology & Microbiology, Faculty of Medicine, American University of Beirut, Riad El-Solh, Beirut, Lebanon.

^3^ Department of Pathology & Laboratory Medicine, Faculty of Medicine, American University of Beirut, Riad El-Solh, Beirut, Lebanon.

* Sima Tokajian: [stokjian@lau.edu.lb](mailto:stokjian@lau.edu.lb)

Lebanese American University, Byblos Campos, P.O. Box 36, Lebanon

Tel: +961-9-547262

Fax: +961-9-546262

[stokjian@lau.edu.lb](mailto:stokjian@lau.edu.lb); [tamata.salloum@lau.edu.lb](mailto:tamata.salloum@lau.edu.lb); [sahar.alousi@lau.edu](mailto:sahar.alousi@lau.edu);

[harout.arabaghian@lau.edu](mailto:harout.arabaghian@lau.edu).lb; [gmatar@aub.edu.lb](mailto:gmatar@aub.edu.lb): [garaj@aub.edu.lb](mailto:garaj@aub.edu.lb);

**Supplementary Table 1.** Genomes used for SNPs phylogenetic tree comparison. List of accession number retrieved from GenBank Nucleotide database.

| Accession Number | Strain |
| --- | --- |
| CU928163 | UMN026 |
| NZ_LNHL00000000 | 50579417 |
| LJOJ01000000 | EC-IMP53 |
| AYOP01000001.1 | LAU-EC4 |
| AYOG01000001.1 | LAU-EC5 |
| CP008697 | ST648 |
| CP018121.1 | MRSN346355 |
| CP018103 | MRSN352231 |
| CP018109 | MRSN346595 |
| CP018115 | MRSN346638 |
| CP010172 | H8 |
| CP010157 | D10 |
| GCA_002192275.1 | AR_0114 |
| CP021175 | 5CRE51 |
| AE005174 | Z1002 |
| GCA_002125925.1 | CH611_eco |
| GCA_002201835.1 | AR_0137 |
| AP017610 | 20Ec-P-124 |
| GCA_001677475.1 | 06-00048 |
| GCA_000158395.1 | D9 |
| GCA_001900655.1 | D4 |
| GCA_000971615.1 | CI5 |
| GCA_000471385.1 | M18 |
| GCA_002012045.1 | Ecol_545 |
| GCA_000235125.1 | C1 |
| GCA_000257275.1 | P12b |
| GCA_001860505.1 | Y5 |
| GCA_001721525.1 | MS6198 |
| GCA_002214205.1 | M160133 |
| GCA_001559615.1 | C2566 |
| AP017620 | MRY15-131 |
| GCA_000227625.1 | CE10 |
| GCA_000009565.1 | BL21 |
